# Retraction: Machine Learning-Based Data Extraction Tools in Healthcare: A Systematic Review

**DOI:** 10.7759/cureus.r242

**Published:** 2026-09-09

**Authors:** Zain Khalpey, Matthew Rorvig, Zacharya I Khalpey, Ujjawal Kumar, Feras H Khaliel, Nicholas King

**Affiliations:** 1 Cardiothoracic Surgery, HonorHealth, Scottsdale, USA; 2 Research, Honor Health, Scottsdale, USA; 3 Surgery, University of California, Los Angeles, Los Angeles, USA; 4 Cardiac Surgery, King Faisal Specialist Hospital and Research Centre, Riyadh, SAU; 5 Cardiac Surgery, Heart Center, King Faisal Specialist Hospital and Research Centre, Riyadh, SAU; 6 Cardiology, HonorHealth, Austin, USA

This article has been retracted by the Editor-in-Chief. Following concerns raised by a reader, a post-publication review by the journal found that seven of the 21 studies presented as included in this systematic review cite no source and are described as "internal, unnamed, or representative studies," that two entries are attributed to authors who do not appear in the reference list, and that the characteristics attributed to several cited studies do not match the source publications. The authors were unable to identify the studies in question. The authors confirmed the factual findings set out above and proposed corrections. They disagree with the decision to retract rather than correct the articles.

